# The validity and reliability of a real-time AI-based neck exercise program among young adults in Thailand: evaluation of accuracy and execution time

**DOI:** 10.3389/frai.2026.1776338

**Published:** 2026-05-19

**Authors:** Yadanuch Boonyaratana, Nacha Chondamrongkul, Vitsarut Buttagat, Pattanasin Areeudomwong

**Affiliations:** 1 Physical Therapy Program, School of Integrative Medicine, Mae Fah Luang University, Chiang Rai, Thailand; 2 Research Group on Smart Integrative Medicine & Technology Sustainability, Mae Fah Luang University, Chiang Rai, Thailand; 3 School of Applied Digital Technology, Mae Fah Luang University, Chiang Rai, Thailand

**Keywords:** accuracy rate, AI real-time program, general cervical exercise, neck pain, validity and reliability

## Abstract

Neck pain caused by weakened muscles and decreased strength may be alleviated with exercise. Integrating digital technology, such as artificial intelligence (AI), into neck exercise programs may improve their accuracy and effectiveness. This study aims to evaluate the validity and reliability of an AI-based neck exercise program to measure accuracy and execution time. The method included evaluating the validity and reliability of a real-time AI neck exercise program among 30 healthy participants aged 18–29 years, across five exercises. In the results, the outcome variables included the content validity index (IOC) for exercise accuracy and execution time. Reliability was evaluated using the intraclass correlation coefficient (ICC), and associations between variables were analyzed using Pearson’s correlation coefficient. The content validity assessment indicated high validity of the AI-based neck exercise program, with IOC values between 0.86 and 1.00 across all exercises. Moderate reliability was observed for exercise accuracy (ICC = 0.63) and execution time (ICC = 0.622). Higher exercise accuracy was associated with shorter execution time; however, this relationship should be interpreted with caution, as it does not imply causality.

## 1 Introduction

Neck pain is the most common musculoskeletal problem worldwide, with an estimated 65 million people experiencing neck pain each year (Barbosa et al., 2023). The common clinical features in individuals with neck pain include abnormal neck posture, such as forward-leaning head and rounded shoulders, and changes in the neck muscles, such as tenderness, increased muscle tone, and decreased muscle endurance and strength (Lertsinthai and Dechmark, 2017). Factors that may contribute to neck pain include prolonged use of phones or computers, leading to prolonged neck flexion, which may result in a forward-leaning head posture. Hence, neck pain may lead to anxiety, depression, and sleep problems (Kazeminasab et al., 2022; Lee et al., 2017; Mahmoud et al., 2019). Meanwhile, physical therapy treatments, such as hot packs, ultrasound, and electrical stimulation (Shin and Choi, 2010), may reduce neck pain. In a previous study, Graham et al. (2013) reported that physical therapy tools helped reduce neck pain immediately after treatment in patients with neck muscle soreness. They also found that exercise not only reduced pain but also increased strength and muscle control and improved quality of life (Shin et al., 2020).

Over the past two decades, digital technology has become widespread and rapidly adopted, including mobile apps, social media, wearable devices, and artificial intelligence (AI). E-learning programs have been used to promote exercise and improve health and well-being (Iyamu et al., 2021). Recent advancements in rehabilitation have enabled digital technologies to improve adherence to and monitor home-based exercise. Among the several technologies available, AI development offers unique advantages, including more complex processing, faster data calculation than humans, and the facilitation of personalized interventions (Sumner et al., 2023). Digitalization may play a major role by combining education and exercise therapy, making the use of apps reasonable, and many applications also include study components (Hartmann et al., 2023).

There are guidelines for selecting assessment tools in clinical trials that relate to the accuracy and reliability of important measurement tools (Mylonas et al., 2022; Sukamolson, 2024). Reliability is the degree to which a measurement produces stable and consistent results, so assessing the measurement tool reliability is necessary in clinical settings (Paraskevopoulos et al., 2020). Therefore, a questionnaire was developed for using the AI program in conjunction with general neck muscle exercises provided by experts. The objective of this study was to examine the content validity of a questionnaire developed for the real-time AI neck exercise program for young adults and to assess its reliability.

## 2 Materials and methods

This research was designed as a content validity study conducted with experts in Thailand, and a reliability check was performed with relevant experts. The content the validity check was performed by three experts, including an exercise physical therapy specialist, an information technology (IT) specialist, and a young adult who would use the AI program. A questionnaire was prepared, and the study procedures were explained in detail to all experts. The experts received the questionnaire along with an electronic exercise posture poster (Supplementary Figure 1) and exercise videos to review for 1 week. The questionnaire consisted of two sections: the first section assessed the neck muscle exercises (four questions), and the second section assessed the AI program format (six questions) for the real-time neck exercise AI program (Supplementary Table 1).

### 2.1 Neck exercise AI program

The real-time AI-based cervical exercise program consisted of five movements—chin tuck, chin out, cervical extension, hand-to-head, and chest stretch—adapted from the Modified McKenzie and Kendall exercise protocols (Kong et al., 2017). The exercises were designed to activate deep cervical flexors, improve cervical posture and mobility, enhance isometric stabilization, and reduce anterior muscular tightness. Each session comprised 50 repetitions with 5-s rest intervals and a total duration of approximately 5–10 min. Exercise performances were monitored using an image-based AI motion-analysis system that continuously tracked predefined anatomical landmarks of the head, neck, and upper trunk. Participant movement trajectories were compared with biomechanical reference models, and real-time corrective feedback were provided to minimize compensatory movements and optimize exercise execution.

### 2.2 The AI software platforms and algorithm types

This study conducted a survey to assess user demands and determine the project scope, which included defining the schedule and budget for the design task. The procedure for developing the AI-based neck exercise program software was as follows: planning the structure and architecture of the software system, including workflow and communication among development components; writing and programming software code in accordance with design criteria; and conducting preliminary testing to ensure accuracy and efficiency. System testing was performed to identify and correct flaws, ensuring that the software functioned as expected. Finally, the software system was deployed by giving users control over installation and updating the existing system.

Specifically, posture recognition was implemented using a camera-based computer vision approach that detects and tracks key anatomical landmarks of the head and neck to classify exercise postures in real time. The training dataset consisted of approximately 250 labeled images, collected under controlled conditions and augmented to improve robustness, including internal validation of posture classification accuracy prior to deployment of the system. Environmental factors were addressed by providing standardized instructions regarding camera positioning, distance, and lighting conditions, and the algorithm was designed to tolerate minor variations in illumination and camera angle.

### 2.3 Sample size

During single session, 30 healthy male and female university students including aged 18–29 years old which sample size estimation was informed by reliability outcomes reported in prior studies with comparable designs and was conducted to ensure an acceptable level of statistical power for intraclass correlation coefficient (ICC) analysis, targeting ICC values in the range of 0.95 (*p* = 0.05) (Salmon et al., 2018). This study was conducted in accordance with the Declaration of Helsinki and approved by the Mae Fah Luang University Ethics Committee on Human Research (EC24126-25).

### 2.4 Validity assessment

The questionnaire scores were used to evaluate the real-time AI neck exercise program by calculating the item-objective congruence (IOC), with the following criteria: (+1) “You are sure the question aligns with the objective,” (0) “You are unsure if the question aligns with the objective,” and (−1) “You are sure the question does not align with the objective.”

### 2.5 Reliability assessment

Regarding the accuracy parameters for the AI neck exercises program, when combined with a real-time AI system, values ranged from −0.01 to 1.00. The higher the accuracy value, the closer it is to the target accuracy. This value was displayed by the real-time AI program. Each exercise was measured thrice and averaged for statistical analysis. Watching the time during neck exercises combined with a real-time AI program, which started from the normal position for each neck exercise and stopped when the accuracy reached 0.90 or higher. A researcher was present to guide adjustments to the exercise positions to achieve the desired accuracy. Each position was measured thrice and averaged for statistical analysis.

### 2.6 Statistical analysis

The researcher collected data for analysis using IBM SPSS Statistics version 20, which included both descriptive and inferential statistics. The mean and standard deviation were used to calculate and present demographic data such as age, weight, height, and BMI. Sex was presented as frequency and percentage values. To calculate the content validity of the questionnaire with the AI program for general neck muscle exercises, the mean (SD) was used to determine the content validity of the IOC, and a graph was created using Microsoft Excel. Therefore, an IOC value greater than 0.5 was considered acceptable. The reliability calculations included the accuracy rate for performing general neck muscle exercises and the time taken to perform general neck muscle exercises, and were assessed based on the average of three repetitions of general neck muscle exercises.

The ICC statistic was used to analyze reliability. The variance was set at 0.05, and the ICC value was defined as >0.90. Data normality was determined using the Shapiro–Wilk test to examine the correlation between the accuracy of performing general neck muscle exercises at the beginning and end of the exercise and the time taken to perform them. Additionally, for the normally distributed data (parametric), the Pearson’s correlation analysis was used. Hence, there was non-normally distributed data (non-parametric), and the Spearman’s correlation coefficient analysis was used, considering both the level of relationship and statistical significance.

## 3 Results

### 3.1 Validity

A content validity study was conducted with three experts in Thailand (validity) using a questionnaire. The questionnaire consisted of two sections. Section 1 was the neck muscle exercise posture evaluation, comprising four questions, and section 2 was the AI program format evaluation, with six questions for each of the five exercise postures in the real-time neck exercise AI program, totaling 50 questions. The questionnaire scores were used to evaluate the real-time neck exercise AI program by calculating the IOC.

**Figure 1:**
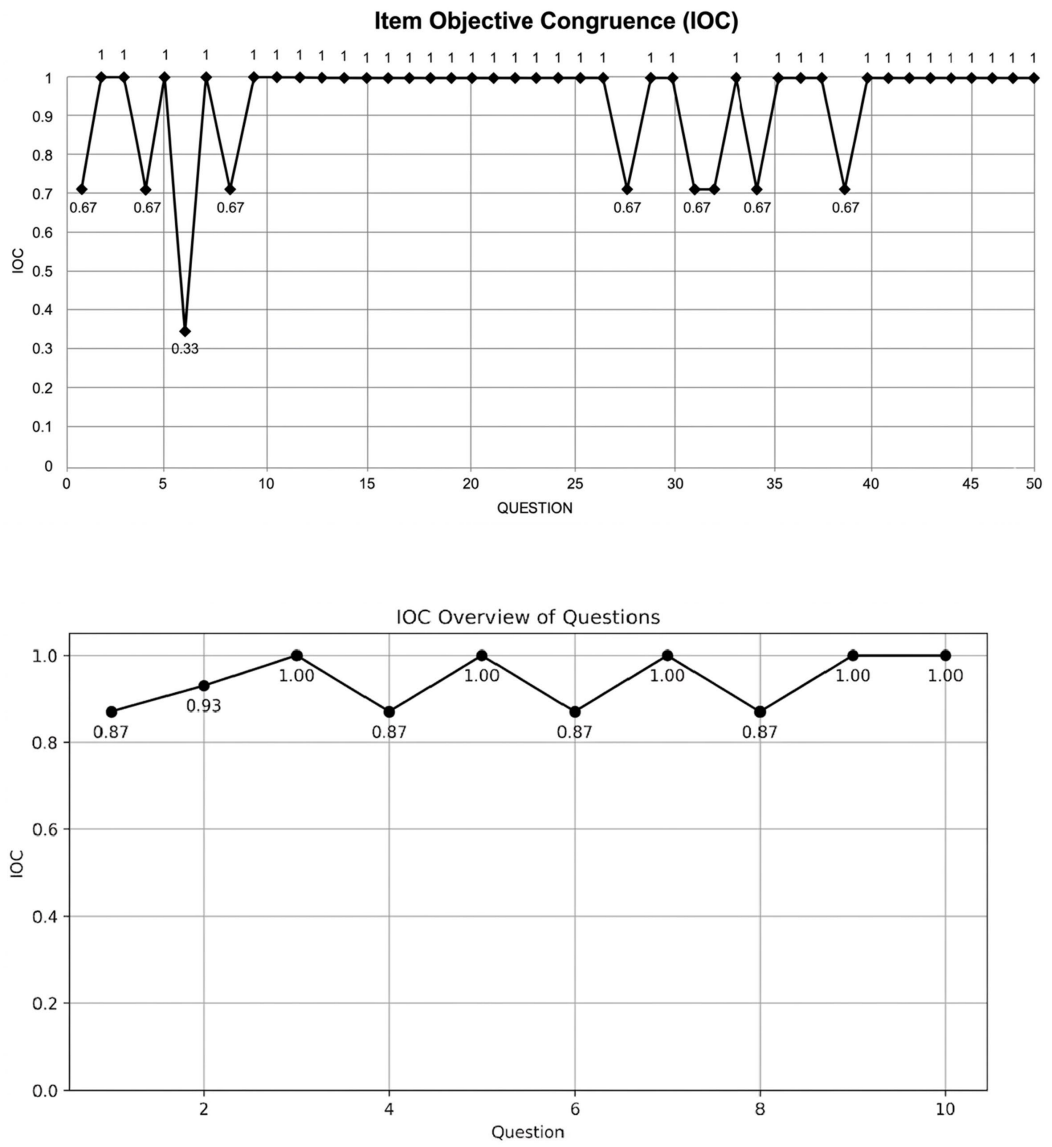
Validity demonstrates for the AI neck exercise program by all three experts.

As shown in Figure 1, validity was demonstrated for the AI neck exercise program by all three experts. They evaluated the questionnaires and provided suggestions for each item. The validity of each item showed IOC = 0.67–1.00, which indicates quite high validity for this program’s exercises. Eight questions showed moderate validity: questions 1, 8, 4, 28, 31, 32, 34, and 38 (IOC = 0.67). However, only the sixth question showed a lower IOC (0.33) due to wording ambiguity; this item was revised prior to reliability analysis and therefore did not affect subsequent results.

### 3.2 Reliability

Descriptive analysis of the participant group indicated that age (years), body weight (kg), height (m), and body mass index (BMI; kg/m^2^) were approximately normally distributed (Table 1). Furthermore, gender was analyzed as a categorical variable and presented as frequencies and percentages. Among the 30 participants, 8 were male (26.7%), and 22 were female (73.3%). No significant differences were observed in the general characteristics of the participants. Continuous variables are reported as mean ± standard deviation, with values of 21.73 ± 1.08 years for age, 69.13 ± 16.66 kg for body weight, 1.65 ± 0.06 m for height, and 25.39 ± 5.18 kg/m^2^ for BMI (Supplementary Tables 2, 3).

**Table 1:** Showed the characteristics data with mean (SD) and percentages.

Characteristic	*N*	%
Age		
Mean = 21.73, S.D. = 1.08		
Gender		
Male	8	26.7
Female	22	73.3
Weight (kg)		
Mean = 69.13, S.D. = 16.66		
Height (meters)		
Mean = 1.64, S.D. = 0.06		
BMI (kg/meter^2^)		
Mean = 25.38, S.D. = 5.18		

Additionally, reliability analysis of the AI-based neck exercise program is presented in Tables 2, 3. According to the classification proposed by Koo and Li (2016), ICC values between 0.50 and 0.75 indicate moderate reliability. In the present study, the ICC for exercise accuracy was 0.63, and the ICC for execution time was 0.622, both corresponding to moderate reliability. These results demonstrate consistent measurement performance of the AI system, although reliability did not reach the thresholds for good or excellent reliability.

**Table 2:** The ICC of accuracy rate with AI neck exercise progarm upon compltetion.

	Intraclass correlation coefficient						
	Intraclass correlation^b^	95% confidence interval		*F* test with true value 0			
		Lower bound	Upper bound	Value	df1	df2	Sig
Single measures	0.102^a^	0.048	0.200	3.842	29	406	0.000
Average measures	0.631^c^	0.432	0.789	3.842	29	406	0.000

Two-way mixed effects model where people effected are random and measures effects are fixed.

a The estimator is the same, whether the interaction effects is present or not.

b Type A intraclass correlation coefficients using and absolute agreement definition.

c This estimate is computed assuming the interaction effect is absent because it is not estimate otherwise.

**Table 3:** The ICC for the execution time of general neck muscle exercise.

	Intraclass correlation coefficient						
	Intraclass correlation^b^	95% confidence interval		*F* test with true value 0			
		Lower bound	Upper bound	Value	df1	df2	Sig
Single measures	0.099^a^	0.043	0.200	2.838	29	406	0.000
Average measures	0.622^c^	0.404	0.790	2.838	29	406	0.000

Two-way mixed effects model where people effected are random and measures effects are fixed.

a The estimator is the same, whether the interaction effects is present or not.

b Type A intraclass correlation coefficients using and absolute agreement definition.

c This estimate is computed assuming the interaction effect is absent because it is not estimate otherwise.

## 4 Discussion

A content validity study was conducted using questionnaires with experts to examine the reliability of an AI program for real-time neck exercises for young adults and to verify the content validity of the questionnaire. This study revealed adequate content validity for the five neck exercises: chin tuck, chin out, cervical extension, hand-to-head, and chest stretch. These exercises were found to be safe, anatomically correct, and effective in stimulating neck muscle function. The real-time neck exercise AI program may be used for neck exercises, considering the ability of the program to accurately and correctly detect neck muscle exercise postures in real time, along with the program’s fast data processing and display capabilities. The program is also convenient to use, as indicated by an IOC value of 0.60–1.00, suggesting a moderate to high level of content validity.

In the reliability study, the accuracy rate for performing general neck muscle exercises and the time taken to perform the exercises had an ICC of 0.63, indicating moderate reliability. The ICC for the time to complete general neck muscle exercises was 0.62, also indicating moderate reliability. The researcher was unable to control environmental influences during the experiment, such as light, color, sound, and the stability of technological equipment, which could affect the accuracy and execution time to complete general neck muscle exercises. Additionally, performance-based measures such as accuracy and execution time may be influenced by learning effects, individual variability, and task complexity, which can reduce reliability estimates compared with more stable physiological or kinematic measures.

This study aligns with the study by Saadati (2023), who examined the reliability and trustworthiness of AI-generated exercise programs, noting that AI is increasingly used in exercise planning but lacks systematic scientific evaluation, which may affect long-term safety, effectiveness, and user trust. To ensure that AI-developed exercise plans are safe and effective, it is crucial to ensure that they are not only technologically stable but also appropriate and beneficial.

According to previous research studies, the development and effectiveness of an AI-powered mobile application to assist with squat exercises, specifically correcting incorrect form, the AI-powered mobile application developed 20,000 video clips from 52 participants performing 400 squats each, resulting in a test accuracy of −0.84 (85%), thereby demonstrating accurate exercise guidance and significant body posture improvements compared with the control group using general videos. Participants using the mobile application showed greater development in knee joint angles and expert scores, indicating the AI system’s ability to detect and evaluate posture in real time. Nevertheless, there were limitations regarding body structure diversity, including height and body shape, and a lack of detailed user-perception data (Chae et al., 2022). Similarly, this study found that the accuracy rate measurements for each exercise were impacted by body structural variety.

The ICC for the accuracy rate in the AI neck exercise program, which was created using about 250 figures, was 0.63, indicating a moderate level of accuracy that did not meet the originally anticipated threshold. However, future studies should focus on adding more figures to improve reliability and raise the accuracy measures to a higher level. A recent study by Yeh and Yang (2025) reported that an AI-based exercise pose recognition and assessment system could identify poses with 99.9% accuracy and movements with 99.0% accuracy. This demonstrates AI’s reliability for real-world environment use, particularly in the context of home exercise for young adults. However, Yeh and Yang (2025) also noted limitations pertaining to environmental impact from behind the camera and camera angles, which could affect the applicability in some situations. This research suggests that AI could increase the accuracy and safety of exercise pose assessment, which aligns with this study’s moderate reliability, given the inability to control environmental influences.

In the experimental results, the time spent exercising correlated positively with the measurement of general neck muscle exercise accuracy and negatively with the time taken to complete the muscle exercises. This is in accordance with the previous findings, which demonstrated that motor practice and skill retention occur both before and after exercise, and after 24 h and 7 days, the exercise group outperformed the control group on skill retention tests. Motor learning theory, which involves stimulating the nervous system through activity and enhancing the function of the hippocampus and motor cortex, may explain the production and maintenance of exercise movements (Jespersen et al., 2023).

The findings of this study could offer valuable insights into improving exercise applications in sports training and physical rehabilitation. However, it was conducted in a single population group, the sample size was small, and data were collected in a room where light and sound were not controlled, allowing external stimuli to enter. It should be noted that disturbances during the program’s execution may have resulted in inaccurate values. In further studies, the sample size should be increased, data collection should be conducted in a controlled environment with stable equipment, and the questionnaire could be developed to be more specific to muscle groups to cover the desired objectives. In particular, the small number of experts may restrict the breadth of perspectives, and future studies should include a larger and more diverse panel of experts to strengthen content validity.

## 5 Conclusion

The study’s content validity and reliability are moderate, which indicates that a real-time AI-based neck exercise program could possibly be applied in a future version. These 5-neck exercise programs are available for those who are interested, and the real-time AI neck exercise program has a respectable dependability value. While exercise accuracy and time spent tended to be positively associated, suggesting that accuracy improved over time, this program might be changed for neck exercises in the future. Eventually, young adults were intentionally selected to establish baseline reliability prior to application in aging populations, where age-related variability may be greater.

## Data Availability

The original contributions presented in the study are included in the article/Supplementary material, further inquiries can be directed to the corresponding author.
