# Supplementary material for: The validity and reliability of a real-time AI-based neck exercise program among young adults in Thailand: evaluation of accuracy and execution time
Source: Front Artif Intell. 2026 May 19;9:1776338. doi: 10.3389/frai.2026.1776338 (PMC13226617; doi:10.3389/frai.2026.1776338)
Supplement: Supplementary file 1 [file Data_Sheet_1.pdf]

Supplementary Material

1 Supplementary Figure

Figure A is all exercises for 5 positions that consist of Chin tuck, Chin out, Cervical extension, Hand to Head and Chest Stretch, respectively.

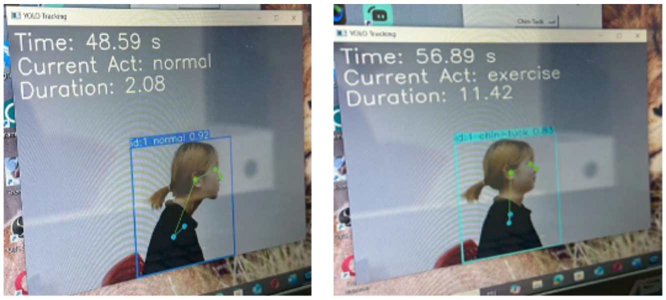

Chin tuck

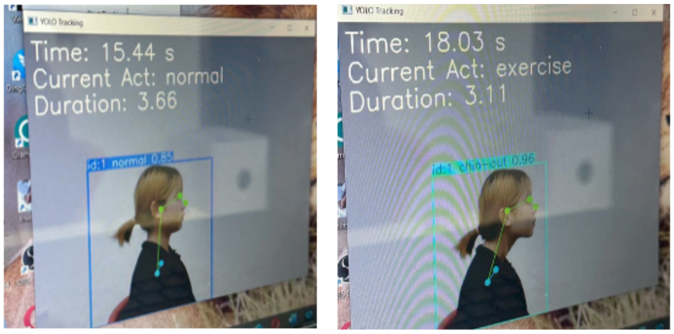

Chin out

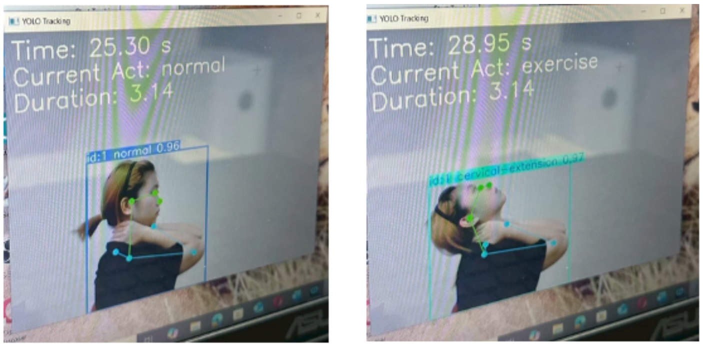

Cervical extension

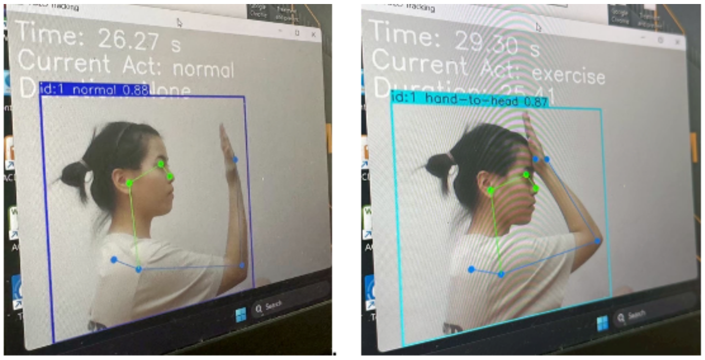

Hand to Head

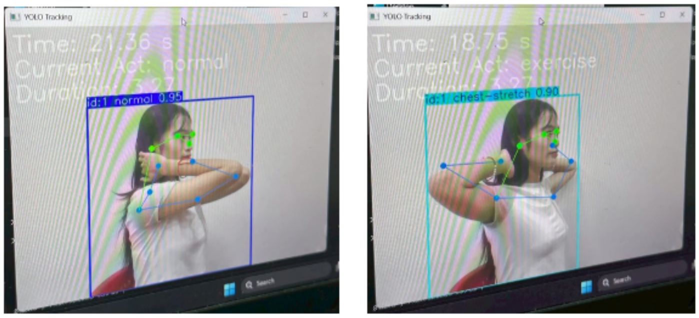

.Chest stretch

2 Supplementary Table

There are tables have shown the correlation coefficient between the accuracy rate of AI neck muscle exercises with the time spent during beginning and ending. The correlation value is set between -0.01 and 1.00, and the significant value at $p < 0.05$ (Table 1-2).

Table 1 shows the correlation between the accuracy rate of general neck muscle exercises at the beginning of the session and the execution time spent.

**Exercise at the beginning point (Accuracy Rate (%))**	**Times spent (sec.) #1**	**Times spent (sec.) #2**	**Times spent (sec.) #3**
	**Correlation(r)/ sig.(2-tailed)**	**Correlation(r)/ sig.(2-tailed)**	**Correlation(r)/ sig.(2-tailed)**
Position 1 at start 1	-0.06/0.75	0.15/0.40	-0.02/0.90
Position 1 at start 2	0.19/0.30	0.15/0.41	0.25/0.17
Position 1 at start 3	0.09/0.61	0.23/0.21	0.23/0.21
Position 2 at start 1	-0.28/0.12	0.01/0.92	0.02/0.88
Position 2 at start 2	-0.18/0.32	-0.07/0.68	-0.05/0.76
Position 2 at start 3	-0.21/0.26	-0.24/0.19	0.00/0.96
Position 3 at start 1	-0.09/0.63	-0.23/0.21	-0.13/0.46
Position 3 at start 2	-0.10/0.59	-0.22/0.23	-0.13/0.49
Position 3 at start 3	-0.26/0.16	-0.31/0.09	-0.21/0.24

Position 4 at start 1	0.11/0.55	-0.19/0.31	-0.15/0.40
Position 4 at start 2	-0.04/0.83	-0.27/0.14	-0.08/0.64
Position 4 at start 3	-0.05/0.78	-0.16/0.38	-0.31/0.09
Position 5 at start 1	0.10/0.56	-0.02/0.90	-0.08/0.64
Position 5 at start 2	0.11/0.55	-0.04/0.82	-0.15/0.41
Position 5 at start 3	0.06/0.71	-0.03/0.87	-0.10/0.59

Table 2: The correlation between accuracy rate of neck muscle exercise and time spent

Exercise at the end point (Accuracy Rate (%))	Times spent (sec.) #1	Times spent (sec.) #2	Times spent (sec.) #3
	**Correlation(r)/ sig.(2-tailed)**	**Correlation(r)/ sig.(2-tailed)**	**Correlation(r)/ sig.(2-tailed)**
Position 1 at stop 1	-0.39*/0.03	-0.36*/0.04	0.30/0.09
Position 1 at stop2	-0.28/0.12	-0.25/0.23	-0.29/0.11
Position 1 at stop3	-0.32/0.08	-0.31/0.09	-0.39*/0.03
Position 2 at stop 1	-0.23/0.21	-0.05/0.77	-0.06/0.73
Position 2 at stop2	-0.24/0.18	0.27/0.88	-0.01/0.94
Position 2 at stop3	-0.26/0.16	0.01/0.94	-0.03/0.86
Position 3 at stop1	-0.189/0.31	-0.18/0.32	-0.10/0.57
Position 3 at stop 2	-0.31/0.07	-0.31/0.09	-0.15/0.42
Position 3 at stop3	-0.32/0.07	-0.32/0.76	-0.21/0.25
Position 4 at stop 1	0.67/0.72	0.27/0.88	-0.22/0.23
Position 4 at stop 2	-0.08/0.66	0.04/0.83	-0.17/0.35
Position 4 at stop3	-0.10/0.58	-0.11/0.54	-0.23/0.20
Position 5 at stop 1	-0.16/0.38	-0.45*/0.01	-0.65**/0.00

Position 5 at stop 2	-0.19/0.30	-0.563**/0.001	-0.633**/0.000
Position 5 at stop3	-0.22/0.24	-0.63**/0.00	-0.67**/0.00
