# Supplementary material for: The validity and reliability of a real-time AI-based neck exercise program among young adults in Thailand: evaluation of accuracy and execution time
Source: Front Artif Intell. 2026 May 19;9:1776338. doi: 10.3389/frai.2026.1776338 (PMC13226617; doi:10.3389/frai.2026.1776338)
Supplement: Supplementary file 2 [file Data_Sheet_2.pdf]

Table 1: a **clear, publication-ready example table** of an **expertise checklist questionnaire** for evaluating an **AI-Neck Exercise Program consisting of 5 exercises (Original version and transtaed version).**

จุดประสงค์ที่/เนื้อหา	ข้อคำถาม	ผลการพิจารณา			ข้อเสนอแนะเพิ่มเติม
		+1	0	-1	
1.ประเมินท่าออกกำลังกายกล้ามเนื้อคอ	1. ท่าออกกำลังกายมีความปลอดภัยต่ออาสาสมัคร ด้านความปลอดภัยต่อผู้ใช้โปรแกรม(ผู้สูงอายุ) โดยไม่ก่อให้เกิดความเสี่ยงในการบาดเจ็บต่อกล้ามเนื้อคอ				
	2. ท่าออกกำลังกายมีคำอธิบายที่ชัดเจน เข้าใจง่ายสะดวกต่อการเรียนรู้และปฏิบัติตามแบบเรียลไทม์				
	3. ท่าออกกำลังกายมีการจัดท่าทางที่ถูกต้องตามหลักสรีระร่างกายมนุษย์และชีวกลศาสตร์ในการทำงานของกล้ามเนื้อ				
	4. ท่าออกกำลังกายในโปรแกรมนี้ก่อให้เกิดเกร็งตัวของกล้ามเนื้อคอ				
2.ประเมินรูปแบบโปรแกรม artificial intelligence (AI)	5. โปรแกรมนี้สามารถตรวจจับท่าทางของการออกกำลังกายกล้ามเนื้อคอที่แม่นยำและถูกต้องแบบเรียลไทม์				
	6. โปรแกมสามารถประมวลผลและแสดงผลข้อมูลได้อย่างรวดเร็วและชัดเจนความสเถียรของโปรแกรมเช่นโปรแกรมสามารถทำงานได้อย่างต่อเนื่องโดยไม่ค้างการทำงาน				
	7. ความสเถียรของโปรแกรม เช่น โปรแกรมสามารถทำงานได้อย่างต่อเนื่องโดยไม่ค้างการทำงาน				

	8.โปรแกรมนี้มีระบบป้องกันปัจจัยต่างๆ ที่อาจรบกวนจากจากสิ่งแวดล้อม เช่น สี,แสง และ เสียง เป็นต้น				
	8. โปรแกรมนี้มีความสะดวก ง่าย ต่อการใช้งานในการออกกำลังกาย				
	10.ความพึงพอใจโดยภาพรวมในการใช้โปรแกรม				

Purpose/content	Questions	Score			Comments
		+1	0	-1	
1. Evaluate neck muscle exercise postures	1.The exercise postures were designed to be safe for participants, including older adults, and were not associated with an increased risk of neck muscle injury.				
	2. The exercises were clearly explained and were easy to understand, learn, and follow in real time.				
	3. The exercise postures were anatomically correct and aligned with established principles of human biomechanics and muscle function.				
	4. The exercise postures in this program effectively activate and strengthen the neck muscles.				
2. Evaluate artificial intelligence (AI) program formats	5. This program can accurately detect correct neck exercise postures in real time.				
	6.The program processes and displays data rapidly and clearly and demonstrates stable performance, allowing continuous operation without system interruption or freezing.				
	7.The program demonstrates stability, enabling continuous operation without system freezing or interruption.				
	8.The program incorporates mechanisms to minimize interference from environmental factors, such as lighting conditions, color variations, and ambient noise.				
	9. The program is convenient and easy to use during exercise.				
	10.Overall satisfaction in using the program				

Table 2: shows the correlation between the accuracy rate of general neck muscle exercises at the start of the session and the execution time. The analysis used a correlation threshold of < –0.90 with statistical significance set at $p < 0.05$.

**Position at start point (Accuracy Rate (%))**	**Times (sec.) #1**	**Times (sec.) #2**	**Times (sec.) #3**
	**Correlation(r)/sig.(2-tailed)**	**Correlation(r)/sig.(2-tailed)**	**Correlation(r)/sig.(2-tailed)**
Position 1 at start 1	-0.06/0.75	0.15/0.40	-0.02/0.90
Position 1 at start 2	0.19/0.30	0.15/0.41	0.25/0.17
Position 1 at start 3	0.09/0.61	0.23/0.21	0.23/0.21
Position 2 at start 1	-0.28/0.12	0.01/0.92	0.02/0.88
Position 2 at start 2	-0.18/0.32	-0.07/0.68	-0.05/0.76
Position 2 at start 3	-0.21/0.26	-0.24/0.19	0.00/0.96
Position 3 at start 1	-0.09/0.63	-0.23/0.21	-0.13/0.46
Position 3 at start 2	-0.10/0.59	-0.22/0.23	-0.13/0.49
Position 3 at start 3	-0.26/0.16	-0.31/0.09	-0.21/0.24
Position 4 at start 1	0.11/0.55	-0.19/0.31	-0.15/0.40

Position 4 at start 2	-0.04/0.83	-0.27/0.14	-0.08/0.64
Position 4 at start 3	-0.05/0.78	-0.16/0.38	-0.31/0.09
Position 5 at start 1	0.10/0.56	-0.02/0.90	-0.08/0.64
Position 5 at start 2	0.11/0.55	-0.04/0.82	-0.15/0.41
Position 5 at start 3	0.06/0.71	-0.03/0.87	-0.10/0.59

The table has shown the correlation coefficient between the accuracy rate of common neck muscle exercises (Accuracy rate) upon completion and the time taken to perform common neck muscle exercises (Time), the correlation value is set between -0.01 and 1.00, and the Sig. value is determined. At $p < 0.05$ (Table 1).

Table 3: the correlation coefficient between accuracy rate of general neck muscle exercise (at completion) and time to perform general neck muscle exercise

**Position at end point (Accuracy Rate (%))**	**Times (sec.) #1**	**Times (sec.) #2**	**Times (sec.) #3**
	**Correlation(r)/sig.(2-tailed)**	**Correlation(r)/sig.(2-tailed)**	**Correlation(r)/sig.(2-tailed)**
Position 1 at stop 1	-0.39*/0.03	-0.36*/0.04	0.30/0.09
Position 1 at stop2	-0.28/0.12	-0.25/0.23	-0.29/0.11
Position 1 at stop3	-0.32/0.08	-0.31/0.09	-0.39*/0.03
Position 2 at stop 1	-0.23/0.21	-0.05/0.77	-0.06/0.73
Position 2 at stop2	-0.24/0.18	0.27/0.88	-0.01/0.94
Position 2 at stop3	-0.26/0.16	0.01/0.94	-0.03/0.86
Position 3 at stop1	-0.189/0.31	-0.18/0.32	-0.10/0.57
Position 3 at stop 2	-0.31/0.07	-0.31/0.09	-0.15/0.42
Position 3 at stop3	-0.32/0.07	-0.32/0.76	-0.21/0.25
Position 4 at stop 1	0.67/0.72	0.27/0.88	-0.22/0.23

Position 4 at stop 2	-0.08/0.66	0.04/0.83	-0.17/0.35
Position 4 at stop3	-0.10/0.58	-0.11/0.54	-0.23/0.20
Position 5 at stop 1	-0.16/0.38	-0.45*/0.01	-0.65**/0.00
Position 5 at stop 2	-0.19/0.30	-0.563**/0.001	-0.633**/0.000
Position 5 at stop3	-0.22/0.24	-0.63**/0.00	-0.67**/0.00
